# The Impact of Winter Months on Venous Thromboembolism (VTE) Patients: A Retrospective Analysis of Hospital Outcomes in the United States

**DOI:** 10.7759/cureus.29091

**Published:** 2022-09-12

**Authors:** Michael Styler, Sachi Singhal, Konstantine Halkidis, Parshva Patel, Kristine M Ward, Maneesh Jain

**Affiliations:** 1 Department of Bone Marrow Transplant and Cellular Therapies, Fox-Chase Temple University, Philadelphia, USA; 2 Internal Medicine, Crozer-Chester Medical Center, Upland, USA; 3 Department of Hematological Malignancies and Cellular Therapeutics, The University of Kansas Medical Center, Kansas City, USA; 4 Department of Internal Medicine, Methodist Medical Center, Oak Ridge, USA; 5 Department of Hematology/Oncology, University of Pennsylvania, Philadelphia, USA; 6 Department of Hematology/Oncology, George Washington University, Washington DC, USA

**Keywords:** national inpatient sample database, outcomes, mortality, winter months, seasonal variation, pulmonary embolism, deep vein thrombosis (dvt)

## Abstract

Objective: We aimed to analyze the Health Care Utilization Project’s (HCUP) Nationwide Inpatient Sample (NIS) and compare mortality rates in hospitals by month to determine if there is seasonal variability in outcomes associated with venous thromboembolism (VTE).

Methods: The Nationwide Inpatient Sample database was queried from 1998 to 2011. Inclusion criteria were a diagnosis of deep vein thrombosis (DVT) (ICD-9 {International Classification of Diseases, Ninth Revision, Clinical Modification} 453.4, 453.8) and/or VTE (ICD-9 415.1) in patients aged 18 years or more. Admission data was then analyzed to compare mortality rates in teaching and non-teaching hospitals over that time and by month. Demographics, Charlson Comorbidity Index, length of stay (LOS), hospital region, and admission types (emergent/urgent versus elective admissions) were assessed. Linear and logistic models were generated for complex survey design to analyze predictors of mortality and LOS.

Results: A total of 1,449,113 DVT/VTE cases were identified in the Nationwide Inpatient Sample (weighted n= 7,150,613), 54.7% female, 56.38% white, 49% in teaching hospitals. Higher mortality was found in the months of November 6.52%, December 6.9%, January 6.94%, and February 6.93% versus overall mortality of 6.4% over 12 months. Higher mortality was noted in these winter months in all regions, along with a significantly increased LOS. Mortality in the total cohort was found to be higher in January, with odds ratio (OR) 1.11 (1.08-1.15), p<0.0001; February, OR 1.11 (1.07-1.15), p<0.0001; and December, OR 1.10 (1.06-1.14), p<0.0001 compared to June. Mortality was significantly lower in the Midwest or North Central regions (OR 0.78 {0.72-0.83}, p<0.0001) and West (OR 0.80 {0.73-0.87}, p<0.0001) compared to the Northeast. Mortality was also significantly higher in teaching hospitals than in non-teaching hospitals (OR 1.16 {1.10-1.22}, p<0.0001), with mortality trending higher in teaching hospitals each month. Emergent/urgent admission, larger hospital size, female sex, age, and urban location were also significantly associated with increased mortality.

Conclusions: This national study identified an increased risk of mortality associated with hospitalizations for DVT/VTE in the winter months, independent of hospital teaching status or region.

## Introduction

Venous thromboembolism (VTE) is a significant cause of morbidity and mortality and complicates the management and life expectancy of hospitalized patients [1]. The incidence of VTE has not changed significantly in the United States in the last three decades and continues to contribute significantly to in-hospital mortality, despite improvements in treatments and prophylactic measures [2-14]. Some studies have suggested that both the incidence of VTE and mortality associated with VTE follow seasonal trends, but the literature is divided. Additionally, the studies to date have been largely single-center, and fewer still include patients hospitalized in the United States [15-24]. One exception to this relative dearth of large-scale analysis includes a study that examined outcomes in patients diagnosed with VTE from 1979-1999 in over 400 hospitals in the United States; the authors found no statistically significant difference in seasonal mortality rates regardless of national region [25]. However, a significant body of literature regarding seasonal effects on the mortality rate of hospitalized patients exists, with results suggesting a general increase in mortality rates in winter months [26-58].

Fluctuations in mortality have been ascribed to several factors. Weather patterns have been implicated, with extremes of weather, either excess cold or excess heat, associated with higher mortality rates [31,32,37,39-45,47,50,53,55-59] One study describes the phenomenon of a “July effect”, showing mortality in patients with VTE to be higher in the summer months. This was attributed to new hospital personnel starting their medical careers at that time of the year, being relatively improperly trained and/or supported to prevent mortality from VTE in this context [60]. Some studies suggest that atmospheric changes over the course of the year may have a role in the incidence of VTE, but the evidence does not appear to support significant variation in the seasonal mortality rate in these patients [15,61].

We sought to determine whether the mortality rates in patients hospitalized in the United States with VTE vary by season from an updated data set compared to that analyzed in the literature to date. Since the clinical landscape in the treatment and prevention of thromboembolic events has changed significantly in recent years, such a study may help clarify whether there are modifiable factors that can be altered to improve mortality rates in patients with VTE in modern United States hospitals.

## Materials and methods

The Nationwide Inpatient Sample (NIS) of Healthcare Cost And Utilization Project (HCUP), A Federal-State-Industry Partnership In Health Data, Sponsored by the Agency for Healthcare Research and Quality (AHRQ) was used to analyze trends in DVT/VTE hospitalization. The NIS database was created for the Healthcare Cost and Utilization Project (HCUP) and contains discharge data from approximately 7 to 8 million discharges per year in the United States, a data set designed to approximate a 20% stratified sample of United States community hospitals, which include those classified as nonfederal, short-term, general and specialty in nature. Estimates are generated using sampling weights provided by the AHRQ. To ensure the internal validity of the NIS, annual data quality assessments are performed. External validity is performed by comparing the NIS data to other hospitalization discharge databases in the United States. This database was queried from 1998-2011 for this study. Discharge level information investigated included patient characteristics like age, sex, race, and insurance status; hospital characteristics like location, teaching vs non-teaching, and bed size; admission characteristics like the length of stay and total charges. For this study diagnosis of VTE was identified by the International Classification of Diseases, Ninth Revision, Clinical Modification (ICD-9-M) codes. Primary diagnosis codes for DVT (ICD-9 453.4, 453.8) and/or VTE (ICD-9 415.1) were used as inclusion criteria. Patients' exclusion criteria included patients aged < 18 years.

The primary outcome analyzed was in-hospital mortality by month. NIS variables were utilized to stratify the cohort by demographic characteristics. The Deyo modification of the Charlson co-morbidity index (CCI) was used to define the severity of co-morbid conditions. The index contains 17 co-morbid conditions weighted differently. CCI scores range from 0 to 33, with greater scores corresponding to a higher burden of co-morbid disease. Hospitals were divided into teaching and non-teaching designations; teaching hospitals were defined as having an American Medical Association-approved residency program, membership in the Council of Teaching Hospitals, or with an equivalent intern and resident-to-patient ratio of >0.25.

Other outcomes included in the analysis were the length of hospital stay (LOS), hospital region (Northeast, South, Midwest or North Central, and West), median household income category based on patients’ zip codes, primary payer (Medicare, Medicaid, private payer including HMO {health maintenance organization}, and self-pay/no charge/other), hospital bed size (small, medium and large), hospital location (rural or urban), admission types (emergent/urgent or elective admission) and disposition at discharge (home, death, or facility/other). Linear and logistic models were generated for complex survey design to analyze predictors of mortality and LOS.

SAS 9.3 (SAS Institute Inc., Cary, North Carolina) was used for analyses. To produce a nationally representative estimate of the entire US population of hospitalized patients, weighted values of patient-level observations were generated. Categorical variables were displayed as percentages and continuous variables as mean values +/- standard error. Chi-square was used to assess differences in groups of categorical variables and a t-test was used for groups of continuous variables. Surverylinear and Surveylogistic models were generated for complex survey design to analyze predictors of mortality and length of stay (LOS). Different multivariate models were used to evaluate odds ratios for mortality in each month.

## Results

Patient-level and hospital-level data for hospital admissions with a diagnosis of DVT or VTE are displayed in Tables 1-2. The number of admissions per month in the cohort is sorted by month of admission, age, sex, race, primary payer for the admission, hospital bed size, hospital status as either non-teaching or teaching, hospital location, and hospital region.

**Table 1:** Patient level variables by month in the cohort. Numbers represent the percentage of patients in the cohort sorted by age, sex, race, and primary payer for the time period of 1998-2011.

Patient Level Variables												
	January	February	March	April	May	June	July	August	September	October	November	December
Age, %												
18-34	6.08	5.98	6.10	6.02	6.41	6.48	6.71	6.79	6.68	6.33	6.31	5.96
35-49	14.39	14.32	14.33	14.71	14.72	15.11	15.42	15.37	15.17	14.80	14.65	14.40
50-64	24.42	24.48	24.53	24.51	24.46	24.62	25.05	25.26	24.81	24.64	24.88	24.42
65-79	32.69	32.74	32.88	32.60	32.62	32.38	31.68	31.70	32.34	32.70	32.49	32.54
>=80	22.41	22.48	22.16	22.15	21.80	21.41	21.14	20.88	21.00	21.54	21.67	22.68
Sex, %	January	February	March	April	May	June	July	August	September	October	November	December
Male	44.77	45.07	44.91	44.81	45.16	45.30	45.77	45.67	45.63	45.35	45.45	45.60
Female	55.23	54.93	55.09	55.19	54.84	54.70	54.23	54.33	54.37	54.65	54.55	54.40
Race	January	February	March	April	May	June	July	August	September	October	November	December
White	56.63	56.63	56.73	56.21	56.37	56.46	56.33	56.02	56.25	56.09	56.51	56.31
Black or African American	12.03	12.04	11.89	12.21	12.24	12.12	12.36	12.37	12.29	12.04	11.95	11.98
Hispanic or Latino	4.35	4.35	4.40	4.51	4.43	4.53	4.58	4.61	4.58	4.49	4.42	4.37
Other	2.88	2.83	2.86	2.84	2.95	2.87	2.88	2.86	2.93	2.97	3.07	3.02
Missing	24.11	24.15	24.13	24.23	24.01	24.02	23.85	24.15	23.95	24.41	24.04	24.31
Primary Payer	January	February	March	April	May	June	July	August	September	October	November	December
Medicare	56.78	56.75	56.87	56.57	56.42	55.73	54.83	54.80	55.37	56.01	55.88	56.89
Medicaid	8.35	8.10	8.39	8.69	8.60	8.64	8.98	8.80	8.68	8.57	8.36	8.32
Private including HMO	28.66	28.84	28.52	28.65	28.54	28.89	29.47	29.64	29.28	28.89	29.01	28.21
Self pay/no charge/other	6.21	6.31	6.22	6.10	6.43	6.74	6.72	6.76	6.66	6.53	6.75	6.58

**Table 2:** Hospital level variables by month in the cohort. Numbers represent the percentage of admissions in the cohort according to hospital bed size, hospital teaching status, hospital location, and hospital region.

Hospital Level Variables															
Hospital bed size	January	February		March	April	May	June	July	August	September	October	November		December	
Small	12.29	12.33		12.23	11.87	12.22	11.90	11.96	11.79	11.78	11.90	11.99		11.77	
Medium	23.99	24.48		24.38	24.28	24.08	24.54	24.09	24.24	24.14	24.29	24.03		24.25	
Large	63.72	63.18		63.40	63.85	63.69	63.56	63.95	63.97	64.08	63.81	63.98		63.98	
Hospital Teaching Status	January	February		March	April	May	June	July	August	September	October	November		December	
Non-Teaching	51.20	51.11		51.13	50.95	51.03	51.06	50.85	50.69	50.91	50.93	50.73		50.85	
Teaching	48.80	48.89		48.87	49.05	48.97	48.94	49.15	49.31	49.09	49.07	49.27		49.15	
Hospital Location	January	February		March	April	May	June	July	August	September	October	November		December	
Rural	13.14	13.05		13.32	12.92	12.96	12.99	12.99	12.92	12.81	12.69	12.76		13.05	
Urban	86.86	86.95		86.68	87.08	87.04	87.01	87.01	87.08	87.19	87.31	87.24		86.95	
Hospital Region	January	February		March	April	May	June	July	August	September	October	November		December	
Northeast	21.85	21.90		21.98	21.85	22.00	21.91	21.93	21.72	21.73	21.73	21.92		21.72	
Midwest or North Central	27.57	27.52	27.47		27.69	27.57	27.67	27.51	27.68	27.45	27.66	27.59	27.68		
South	31.50	31.66	31.68		31.65	31.29	31.63	31.61	31.64	31.69	31.67	31.43	31.30		
West	19.08	18.92	18.87		18.80	19.14	18.79	18.95	18.96	19.13	18.94	19.05	19.30		

We found that for patients admitted to United States hospitals with VTE, the mortality rate was higher in the winter months (Tables 3-4). Using June as a referent month, the odds ratio (OR) for death was significantly higher for patients who were hospitalized in January (OR 1.11 {95% CI 1.08-1.15}), February (OR 1.11 {95% CI 1.07-1.15}) and December (OR 1.10 {95% CI 1.06-1.14}) (Table 5). A trend toward lower mortality rates was observed in July, August, and September, but this trend was not statistically significant.

**Table 3:** Mortality rate by month in the cohort according to patient level variables of age, sex, race, and primary payer.

Mortality Rate													
Age, %	January	February	March	April	May	June	July	August		September	October	November	December
18-34	2.17	2.46	2.17	2.44	2.21	2.26	2.23	2.38		1.93	2.29	2.06	2.37
35-49	3.63	3.54	3.39	3.44	3.31	3.27	3.08	3.47		2.90	3.38	3.42	3.55
50-64	6.02	5.81	5.38	5.31	5.38	5.41	5.69	5.43		5.27	5.46	6.02	5.99
65-79	8.13	7.92	7.49	7.40	7.45	7.42	7.10	7.04		7.35	7.29	7.34	7.95
>=80	9.65	10.05	8.73	8.90	8.65	8.55	8.36	8.41		8.38	8.93	9.27	9.70
Sex, %	January	February	March	April	May	June	July	August		September	October	November	December
Male	7.31	7.35	6.82	6.57	6.65	6.59	6.25	6.27		6.12	6.46	6.81	6.91
Female	6.65	6.59	5.94	6.15	5.95	5.90	5.92	5.87		5.93	6.16	6.29	6.90
Race	January	February	March	April	May	June	July	August		September	October	November	December
White	7.04	7.10	6.41	6.42	6.49	6.32	6.18		6.13	6.18	6.38	6.62	7.06
Black or African American	7.33	7.19	6.60	6.32	6.30	6.53	6.24		6.16	6.33	6.95	6.88	7.13
Hispanic or Latino	7.51	6.86	6.74	6.35	6.32	5.90	6.43		6.68	6.62	6.30	7.91	7.61
Other	9.21	8.92	8.09	8.64	7.75	8.62	7.52		8.36	8.32	7.61	8.06	8.18
Missing	6.16	6.17	5.74	5.88	5.52	5.55	5.46		5.44	5.06	5.63	5.67	6.14
Primary Payer	January	February	March	April	May	June	July		August	September	October	November	December
Medicare	8.30	8.30	7.54	7.49	7.44	7.33	7.23		7.15	7.26	7.47	7.70	8.21
Medicaid	6.10	6.21	5.54	5.46	4.86	5.77	5.41		5.47	5.27	5.55	5.99	5.67
Private including HMO	4.86	4.81	4.59	4.63	4.66	4.55	4.55		4.55	4.23	4.55	4.79	5.06
Self pay/no charge/other	5.28	5.30	4.52	5.05	4.83	4.63	4.14		4.62	4.45	4.98	4.97	5.15

**Table 4:** Mortality rate by month in the cohort according to hospital level variables of hospital bed size, teaching status, location, and region.

Hospital Level Variables												
Hospital bed size	January	February	March	April	May	June	July	August	September	October	November	December
Small	6.12	6.48	5.73	5.82	5.98	5.90	5.39	5.41	5.51	5.55	5.85	6.08
Medium	6.79	6.92	6.21	6.11	5.93	5.80	5.69	5.95	5.57	5.97	6.16	6.65
Large	7.16	7.01	6.50	6.51	6.44	6.43	6.34	6.21	6.29	6.55	6.78	7.16
Hospital Teaching Status	January	February	March	April	May	June	July	August	September	October	November	December
Non-Teaching	7.38	7.32	6.73	6.69	6.52	6.72	6.61	6.47	6.50	6.90	7.01	7.37
Teaching	6.53	6.55	5.95	5.98	6.01	5.73	5.56	5.65	5.56	5.71	6.04	6.46
Hospital Location	January	February	March	April	May	June	July	August	September	October	November	December
Rural	5.93	6.23	5.60	5.54	5.53	5.13	5.13	5.23	5.09	5.30	5.60	5.89
Urban	7.10	7.03	6.45	6.45	6.37	6.38	6.21	6.18	6.16	6.44	6.66	7.06
Hospital Region	January	February	March	April	May	June	July	August	September	October	November	December
Northeast	7.77	7.86	7.24	7.12	7.18	7.09	6.75	6.99	6.84	7.36	7.49	7.91
Midwest or North Central	6.00	5.93	5.36	5.33	5.27	5.37	5.26	5.13	5.01	5.41	5.57	5.85
South	7.21	7.14	6.57	6.54	6.45	6.28	6.32	5.95	6.10	6.31	6.64	7.10
West	6.94	6.96	6.29	6.57	6.32	6.29	6.03	6.52	6.38	6.34	6.60	6.96

**Table 5:** Odds ratio for patient mortality in the cohort. June is used as the referent month. Months in which the odds ratio is statistically significant are in bold.

Admission Month	OR	95% CI Lower Limit	95% CI upper Limit	P-value
January	1.11	1.08	1.15	<0.001
February	1.11	1.07	1.15	<0.001
March	1.02	0.98	1.05	0.41
April	1.01	0.97	1.05	0.69
May	1.00	0.96	1.03	0.89
June	Referent	Referent	Referent	Referent
July	0.99	0.95	1.02	0.47
August	0.98	0.94	1.01	0.22
September	0.97	0.93	1.01	0.11
October	1.01	0.97	1.04	0.72
November	1.04	1.00	1.08	0.06
December	1.10	1.06	1.14	<0.001

**Figure 1:**
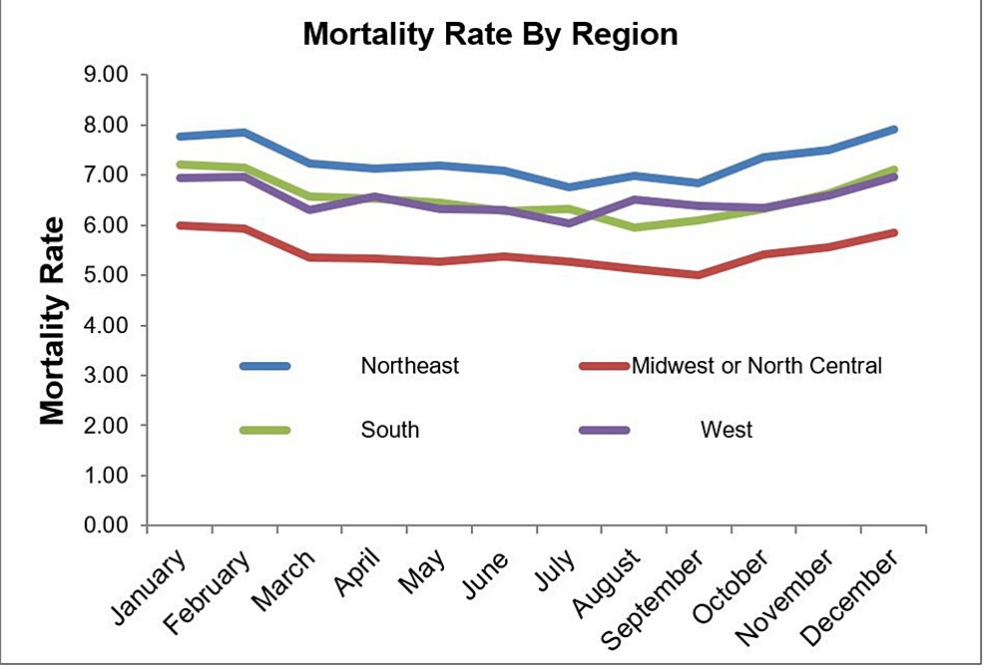
Graphic depiction of the percentage of patient deaths per month in the cohort studied and stratified by regions (Northeast, Midwest or North Central, South, West).

An increased mortality rate in January, February, and December was observed in each region of the country (Tables 4-5, Figure 1). The greatest difference in mortality rates between the referent month of June and the winter months was found in the South region, with odds ratios of death in January, February, and December of 1.16, 1.15, and 1.14, respectively (Table 4).

Comparing different geographical regions in the United States, the lowest mortality from VTE was seen in the Mid-West/North Central region (OR 0.78 {0.72-0.83} p <0.0001), compared to the North East, throughout the year. The highest mortality rates were observed in the North East, and in all regions the mortality rates fluctuated significantly through the months, with the highest being in the winter months and the lowest in July, August, and September. Of note, teaching hospitals had significantly higher mortality from VTE across the months compared to non-teaching hospitals (OR 1.16 {1.10-1.22} p <0.0001).

## Discussion

**Figure 2:**
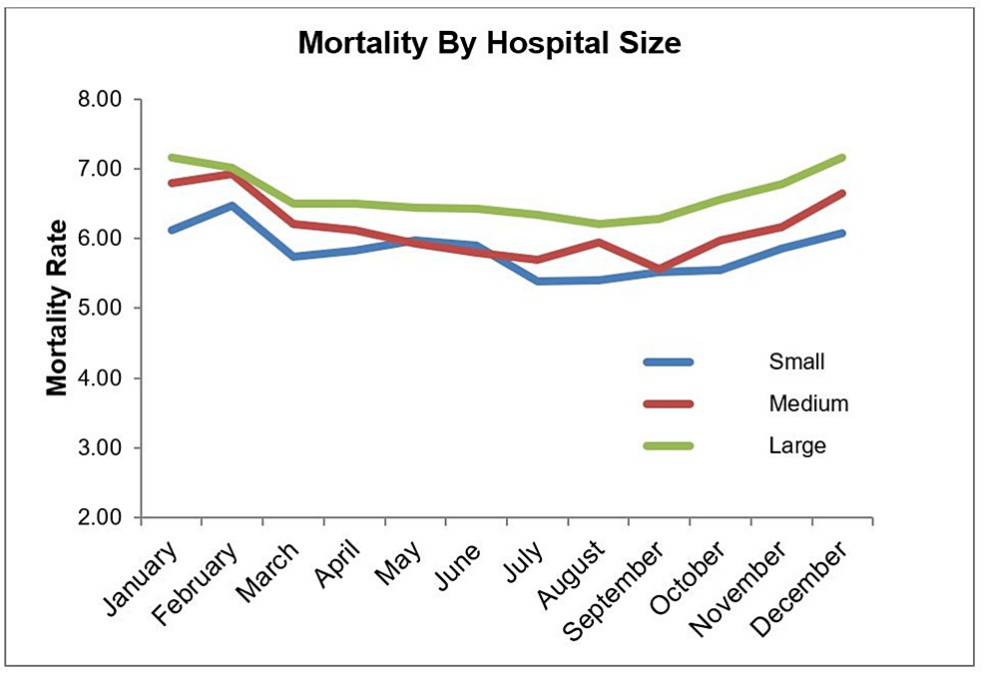
Monthly mortality rate by hospital size.

**Figure 3:**
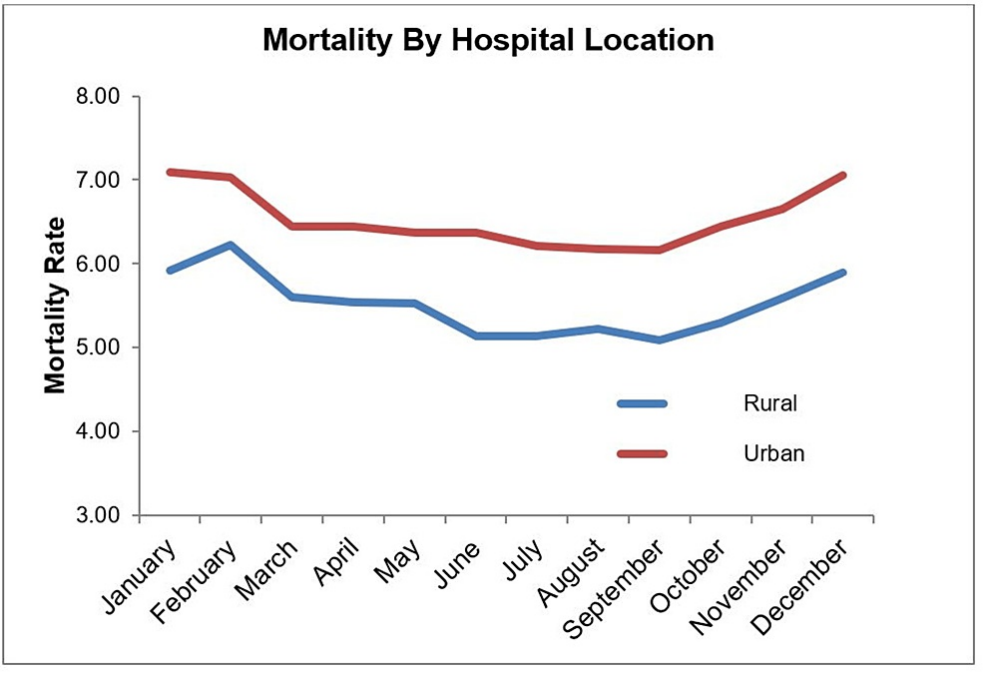
Monthly mortality rate by hospital location.

We found that mortality associated with VTE increases significantly from December to February, despite no significant fluctuations in incidence. The observations reported herein, therefore, likely reflect a general increase in mortality rates at that time of the year in the United States previously reported in the literature [25-59]. The increased mortality rate was observed regardless of the status of the hospitals studied as either teaching or non-teaching (Figure 2); the size of the hospitals studied (Figure 3); or the location of the hospital (either Rural or Urban). The trend toward increased mortality in the winter was observed in every region of the country, including the South region, where lower temperatures are not as extreme. This lack of regional variability correlates with the findings of previous research, but in contrast, we did not observe a lack of seasonal effect on mortality rate [24]. Furthermore, we did not observe an increase in mortality rates in July in any of our sub-group analyses.

The reasons for the increased mortality rate in winter months associated with VTE likely reflect several underlying factors. Some of the potential factors are discussed below. 

Ambient temperatures are generally lower in the United States in the months in which the increase in mortality was observed [40]. These effects are seen in other parts of the world as well. A study from northeastern China demonstrated a similar significant seasonal variation in DVT, with an increased number of hospitalizations for DVT in the winter months [62]. Some of the mechanisms proposed to explain this increased mortality rate include alterations in blood pressure and heart rate associated with lower ambient temperatures [43]. A recent study from South Serbia showed significantly higher creatinine levels in patients developing DVTs in spring, and significantly higher low-density lipoprotein (LDL) levels in patients developing DVTs in winter [63]. However, as expected with a phenomenon as complex as seasonal mortality, no clear causal connection between lower temperatures and increased mortality has been established. Furthermore, we observed that areas with much less temperature variation, i.e., the southern areas, also had an increased mortality rate during winter months. Hence, temperature fluctuations alone are likely inadequate to explain why mortality increases in the winter months.

Alternate weather-related theories as to differences in seasonal mortality rates, specifically regarding VTE, include a small body of literature connecting changes in atmospheric pressure with an increase in both the incidence and mortality of VTE [15,61]. There is some evidence that atmospheric changes in pressure affect the concentration of prothrombin and factor VIIa activity [64]. However, the study cited was designed to reproduce the environment encountered during commercial air travel, which induces far more dramatic changes in the local baro-environment of humans than the daily change of the weather over the course of a year. Li et al. noted high evaporation and high vapor pressures on the date of admission from DVTs [62]. Another small study found that platelet size, fibrinogen levels, and total factor VII levels change with the seasons, but each parameter analyzed peaked at different times of the year respective to one another [65]. The fact that any effect of ambient pressure on hematological parameters was observed suggests a possible relationship between pressure and death in patients with VTE.

Though temperature and atmospheric pressure changes are clearly associated with changes in season, infection rates also vary throughout the year. Notably, the time frame of the increased mortality rate observed in our study coincides with influenza season in the United States [61]. Influenza epidemics invariably lead to an increase in mortality rates [27,41,66-72]. Patients concomitantly suffering from influenza and a VTE may be more likely to die than patients who have either one or the other. However, we lack clear causation and correlation in the existing literature. There are many other infections with a peak incidence in winter, including but not limited to bacterial pneumonia [73]. There is scope for prospective studies that are designed to evaluate an association between infectious disease, VTE, and increased mortality.

Winter months in the northern hemisphere feature shorter days and longer nights. Vitamin D levels have been shown to fluctuate with light levels in multiple studies [73,74]. Currently, research into the association between vitamin D deficiency and adverse outcomes in hospitalized patients is an area of interest [75-79]. The effects of vitamin D deficiency are myriad and affect every system in the body [72]. It evokes the question of a potential connection between lower levels of vitamin D and increased mortality in patients with VTE and needs to be further explored. Potential applications of such studies would probably be most useful on the epidemiological level, as vitamin D levels vary significantly between individuals in the general population [74]. A recent nationwide observational study of approximately 21 million hospitalizations showed that major teaching hospital status was associated with significantly lower mortality rates for common conditions when compared with nonteaching hospitals (8.3% vs 9.5%) [80]. Our data contradicts this observation - one possible explanation is that more complicated and severe cases are usually sent to big referral/teaching centers, but the exact reasons behind these differences are yet to be studied.

Another potential contributing factor to an increased mortality rate in the winter in patients with VTE could be hospital-related factors, such as fluctuations in hospital staff during weekends and holidays. Previous studies into this effect have shown heterogeneous results [81-83]. It remains to be seen if this phenomenon contributes to increased mortality rates in winter months in patients with VTE or if other hospital-related phenomena are partially to blame. Such an analysis as the one we performed could be done to see if specific holiday periods are associated with increased mortality, particularly in the winter months utilizing the same or a similar nationwide database. Additionally, we propose that the rate of clinical depression is likely higher in the winter months, and this fact could affect patients as well as the medical personnel caring for them. This is an area that is underrepresented in the literature and could be a robust area for study in the future.

Our study had limitations associated with administrative claims data, which contains codes produced for billing and documentation purposes. Being a retrospective study, we can only report an association between DVT/PE-associated mortality and months of the year. An in-depth prospective study might be needed to evaluate further potential factors playing a role in this association. Also, our dataset evaluates the population between the years 1998 and 2011, and the lack of data beyond that is a potential limitation of the study. A similarly designed study on a more updated dataset might be beneficial to compare and contrast outcomes over the decades. Since ICD-10-CM codes were used to identify all the diagnoses and associated comorbid conditions, the possibility of coding errors cannot be overlooked.

## Conclusions

In conclusion, we observed a statistically significant increase in mortality in the winter months of November, December, January, and February across all regions of the country, regardless of the teaching and non-teaching status of the hospitals. Although the exact cause is not well understood, a more aggressive DVT prophylaxis regimen can be considered in hospitalized patients through the winter months, and informing the hospital staff of these seasonal fluctuations may help to improve outcomes in patients with DVT.
